# Enhanced In Vitro Stability of Bedaquiline with Ascorbic Acid and Pyruvate During Long-Term Incubation in *Mycobacterium* Species

**DOI:** 10.3390/antibiotics15030316

**Published:** 2026-03-20

**Authors:** Sara Batista, Jordi Lamata, Lidia Feliu, Marta Planas, Mariana Fernandez-Pittol, Diego Martinez, Lorena San Nicolás, Griselda Tudó, Julian Gonzalez-Martin

**Affiliations:** 1 Departament de Fonaments Clínics, Facultat de Medicina i Ciències de la Salut, Universitat de Barcelona, c/Casanova 143, 08036 Barcelona, Spain; 2 Fundació de Recerca Clínic Barcelona-Institut d’Investigacions Biomèdiques Augustí Pi i Sunyer (FRCB-IDIBAPS), 08036 Barcelona, Spain; 3 Servei de Microbiologia, CDB, Hospital Clínic de Barcelona, c/Villarroel 170, 08036 Barcelona, Spain; 4 LIPPSO, Departament de Química, Institut de Tecnologia Agroalimentària, Universitat de Girona, Maria Aurèlia Capmany 69, 17003 Girona, Spainlidia.feliu@udg.edu (L.F.); marta.planas@udg.edu (M.P.); 5 ISGlobal Barcelona, Institute for Global Health, 08036 Barcelona, Spain; 6 CIBER of Infectious Diseases (CIBERINFEC), Instituto de Salud Carlos III, 28029 Madrid, Spain

**Keywords:** bedaquiline, minimum inhibitory concentration (MIC), antibiotic stability, ascorbic acid, pyruvate, *Mycobacterium avium* complex, *Mycobacterium tuberculosis*, antimicrobial susceptibility test

## Abstract

Background: Drug susceptibility testing in *Mycobacterium* species typically requires prolonged incubation periods during which the chemical integrity of antibiotics may not be maintained, potentially compromising the reliability and accuracy of minimum inhibitory concentration (MIC) determinations. Objectives: This study evaluated the in vitro stability of several antibiotics, including recently introduced agents (bedaquiline [BDQ], pretomanid, delamanid and clofazimine) used for treating multidrug-resistant mycobacteriosis (linezolid and moxifloxacin), and those commonly included in combination regimens (rifampicin, isoniazid, ethambutol and clarithromycin). Methods: Antibiotics were pre-incubated at 37 °C before MIC determination and those exhibiting two or more dilutions in MIC were further tested in combination with ascorbic acid (AA) and pyruvate (P). Results: All antibiotics demonstrated stability except BDQ, which showed significant MIC variation after pre-incubation, which was prevented when BDQ was combined with AA and P. Conclusions: These findings suggest that the combined use of AA and P may serve as an effective stabilizing strategy for BDQ during MIC determination.

## 1. Introduction

Tuberculosis is caused by M*ycobacterium tuberculosis* (MTB) and is the leading cause of death due to infectious disease worldwide [1]. Moreover, there has been a notable rise in infections caused by non-tuberculous mycobacteria (NTM) [2,3], particularly those belonging to the *Mycobacterium avium* complex (MAC), with *Mycobacterium avium* and *Mycobacterium intracellulare* being the species most frequently isolated [4,5]. Standard mycobacterial treatment consists of prolonged combination antibiotic therapy [6,7]. According to the World Health Organization, there is around 3.2% of multidrug-resistant tuberculosis (MDR-TB) worldwide [8]. The treatment of MDR-TB commonly includes the combination of bedaquiline (BDQ), pretomanid (PTM), linezolid (LZD) and moxifloxacin (MOX), or even clofazimine (CFZ) [8]. New combination regimens are being explored in vitro to enhance bactericidal efficacy to provide other treatment options that may be less toxic and better tolerated. In these studies, time-kill assays and microdilution methods, which usually require extended incubation periods due to the slow growth rate of mycobacteria, are used [9].

Stability is a critical factor of antibiotic efficacy, especially in assays requiring prolonged incubation, which may lead to antibiotic degradation and affect the measurement of minimum inhibitory concentrations (MIC) and assessment of bactericidal activity [10]. Routine antimicrobial susceptibility testing in *Mycobacterium* spp. does not usually account for antibiotic stability over time. While this aspect has been extensively studied for tigecycline [11], its relevance in other antibiotics remains unclear. Indeed, data on the degradation kinetics of BDQ remain scarce [12] and little is known about its stability and degradation over time, especially under experimental conditions such as time-kill assays. In this context, some studies regarding in vitro antibiotic degradation have been conducted [10,11,13,14] and have reported that factors such as pH, temperature, and light exposure can potentially lead to loss of antibiotic activity over time [10,13,14]. Interestingly, tigecycline showed an exponential loss of activity, which was detained for up to seven days with the addition of ascorbic acid (AA) and pyruvate (P) [11]. The use of AA as a well-established antioxidant has a long history as a protective agent against oxidative stress [15]; P also exhibits antioxidant properties and has been described to exert additive effects when combined with AA [11]. In this study, we specifically investigated BDQ activity during prolonged incubation in an aqueous in vitro environment at 37 °C, a scenario relevant to laboratory drug susceptibility testing but not to clinical formulations.

**Figure 1:**
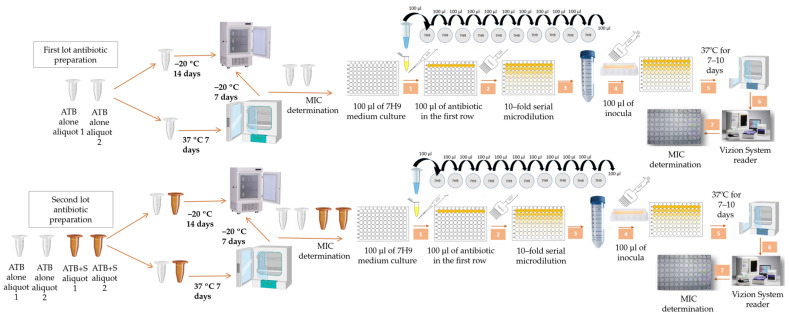
Workflow for antibiotic preparation, pre-incubation, and MIC testing in the absence and presence of stabilizers (ascorbic acid and pyruvate).

The main objective of this study was to evaluate the stability and antimicrobial efficacy of 10 antibiotics, including newer anti-tuberculosis agents (BDQ, PTM, delamanid (DLM) and CFZ) as well as antibiotics used to treat MDR-TB (LZD and MOX) and those included in combination studies (rifampicin (RIF), isoniazid (INH), ethambutol (EMB) and clarithromycin (CLA)), under controlled antibiotic pre-incubation conditions (0 and 7 days at 37 °C) in MTB and MAC clinical isolates (Figure 1). The secondary objective was to evaluate whether AA and P, which have previously shown to stabilize tigecycline [11], can stabilize antibiotics showing a two-fold or greater increase in MIC after pre-incubation at 37 °C.

## 2. Results

All the antibiotics tested in MTB isolates (CFZ, INH, LZD, MOX and PTM) maintained the same or a one-dilution variation in MIC when incubated for 0 days and pre-incubated for 7 days at 37 °C compared to non-pre-incubated samples (Table 1), except for BDQ. A similar result was obtained for the antibiotics tested in MAC isolates (Table 2).

The pre-incubation of BDQ for 7 days at 37 °C demonstrated an increase in MIC by two or more dilutions compared to non-pre-incubated BDQ (0 days). This occurred in both MTB and MAC isolates with MIC ranges increasing from 0.01 to 0.06 µg/mL to 0.06–0.25 µg/mL in MTB isolates, and from 0.03 to 0.125 µg/mL to 0.125–0.5 µg/mL in MAC isolates (Table 3).

Regarding the MAC species, the MICs ranged from 0.03 to 0.125 µg/mL to 0.25–0.5 µg/mL, but were slightly lower in *M. intracellulare*, ranging from 0.03 µg/mL to 0.125–0.25 µg/mL.

When non-pre-incubated (0 days) BDQ was combined with AA and P stabilizers in MTB isolates, the MICs decreased by one dilution compared to the antibiotic without stabilizers, with concentrations ranging from <0.01 to 0.03 µg/mL. In MAC isolates, under the same conditions the MICs remained stable, ranging from 0.03 to 0.125 µg/mL, comparable to those observed in non-pre-incubated samples. The MICs of AA and P were >8 ug/mL, showing no antimicrobial activity of these stabilizers.

Pre-incubation of BDQ with AA and P exhibited either no change in MICs or a one-dilution variation relative to non-pre-incubated BDQ in both MTB and MAC isolates. The difference in reduction in MICs between BDQ pre-incubated without stabilizers and BDQ pre-incubated with AA and P was between three and four dilutions in MTB isolates and between two and three dilutions in MAC isolates.

Replicate measurements performed in triplicate were consistent within the expected assay variability, remaining within one two-fold dilution across all the isolates tested.

**Figure 2:**
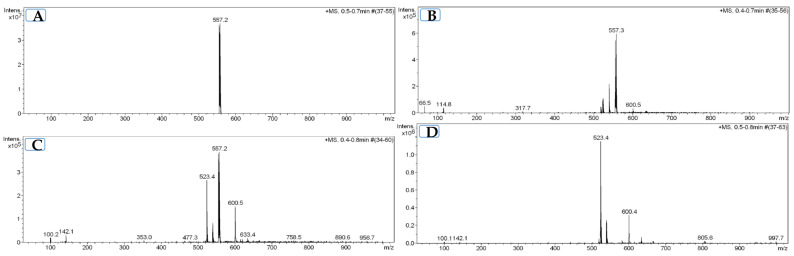
Electrospray ionization mass spectrometry (ESI-MS) profiles of bedaquiline under different incubation conditions. (**A**) BDQ (*m/z* 557) freshly prepared and analyzed after 0 days of incubation at 37 °C. (**B**) BDQ pre-incubated for 7 days at 37 °C. (**C**) BDQ pre-incubated for 7 days at 37 °C with the stabilizers (AA/P). (**D**) BDQ incubated for 7 days at 37 °C with stabilizers and inoculum.

Electrospray ionization mass spectrometry (ESI-MS) analysis of BDQ powder showed a single peak at *m*/*z* 557, corresponding to intact BDQ. The BDQ solution without prior incubation at 37 °C (0-day incubation) displayed the same unique peak at *m*/*z* 557 (Figure 2). In contrast, BDQ solutions pre-incubated for 7 days at 37 °C showed additional peaks at *m*/*z* 523 and *m*/*z* 539, which appeared together with the BDQ peak at *m*/*z* 557. These peaks were also detected when BDQ was pre-incubated under the same conditions in the presence of AA/P.

When BDQ was incubated with bacteria at 37 °C, the peaks at *m*/*z* 523 and *m*/*z* 539 were also observed. Under these conditions, the BDQ peak at *m*/*z* 557 was no longer detected. The appearance of the *m*/*z* 523 and *m*/*z* 539 peaks during bacterial incubation was not prevented by the addition of AA and P.

Antibiotics were either frozen immediately after preparation or incubated at 37 °C for 7 days. Following incubation, the antibiotics were frozen at −20 °C for one week before MIC determination using the microdilution method in 96-well microtiter plates. MIC: minimum inhibitory concentration, ATB: antibiotic, S: stabilizers (ascorbic acid and pyruvate), ATB alone: antibiotic without stabilizers (white), ATB + S: antibiotic with stabilizers (brown).

## 3. Discussion

The primary finding of this study is that BDQ exhibited a loss of antimicrobial activity after 7 days of incubation at 37 °C under aqueous in vitro microdilution conditions. This temperature-dependent loss of activity, observed in both *MAC* and *MTB* clinical isolates, corresponded to an increase of approximately two two-fold dilutions in MIC values, although this shift did not change the overall susceptibility category. In contrast, other anti-tuberculosis agents tested (CFZ, LZD, MOX and PTM) remained stable under the same conditions, highlighting that this phenomenon appears to be BDQ specific.

Extended incubation is inherent to mycobacterial drug-susceptibility testing [6,7,16,17,18] because visible growth requires several days, regardless of the molecular target of a drug. Although BDQ acts on adenosine triphosphate synthase, MIC determinations depend on growth-inhibition endpoints, and the prolonged incubation period reflects the slow growth of *Mycobacterium* spp. Consistent with earlier reports that BDQ can be sensitive to temperature-dependent instability [13], our results confirm that prolonged exposure at 37 °C under assay conditions reduces BDQ activity. This loss of activity can, in turn, lead to inaccurate MIC values, potentially masking the true potency of the drug in vitro.

We initially hypothesized that AA and P, which have previously been shown to stabilize tigecycline activity [11], might counteract BDQ instability. Microbiological results showed that BDQ combined with AA and P retained MIC values similar to freshly prepared BDQ, suggesting an apparent protective effect. The ESI-MS analysis revealed that BDQ undergoes chemical changes at 37 °C, suggesting a loss of BDQ activity detected by the drug susceptibility test. The spectra showed peaks at *m*/*z* 523 and 539 in all samples except for non-pre-incubated BDQ (*m*/*z* 557). These new ions lacked bromine isotope signatures and likely correspond to oxidation products. However, these degradation-associated ions were not prevented by AA and P. Thus, despite the apparent microbiological restoration of activity, the physicochemical data do not support a true stabilizing effect comparable to that described for tigecycline. The role of AA and P remains unclear here, and thus, further studies incorporating stability-indicating high-performance liquid chromatography (HPLC), liquid chromatography coupled to tandem mass spectrometry fragmentation, and, whenever feasible, nuclear magnetic resonance of enriched fractions are necessary to elucidate the underlying mechanisms.

Vilchèze et al. [19] proposed that AA potentiates BDQ activity in MTB by a multifactorial process. The latest findings have suggested synergistic or additive interactions between BDQ and AA in MTB isolates, largely attributed to AA-driven processes, such as oxygen scavenging, increased redox stress, reactive oxygen species generation, and the promotion of dormancy-like physiological states that enhance BDQ susceptibility [15,19,20]. However, our results do not support synergy: the MIC values of BDQ with and without AA/P showed no variation in MAC isolates, while in MTB isolates, they differed by only one dilution, which we do not consider significant. Nevertheless, this modest shift might be consistent with previous reports describing only a limited additive effect of AA on BDQ susceptibility in MTB [15,19,20]. These studies propose that AA can promote bacterial dormancy and alter respiratory metabolism by oxygen scavenging, thereby increasing BDQ susceptibility in MTB isolates; however, such physiological changes are unlikely to occur under the shorter experimental timeframe used here. While dormancy and a true latent state are well-established features of MTB isolates, there is no evidence supporting an equivalent latent or dormant stage in NTM; instead, NTM exhibit a form of functional persistence driven by biofilm formation, slow growth, and intrinsic resistance [21]. The absence of a similar effect in MAC isolates further suggests that the slight MIC reduction in MTB reflects species-specific physiological responses rather than protection of BDQ from degradation. Overall, the increased BDQ activity observed in the presence of AA is more plausibly explained by biological or physicochemical interactions affecting bacterial physiology, rather than by chemical stabilization of BDQ.

Considering the physicochemical properties of this antibiotic, BDQ is a highly hydrophobic compound with poor aqueous solubility, and thus, improved solubility could theoretically increase its bioavailability. A recent study by Godse et al. [22] showed that co-formulating BDQ with AA enhances its dissolution profile, leading to increased permeability and potentially improved antimicrobial activity. Their work also reported strong interactions between BDQ and AA that contributed to stabilizing the drug. However, these findings did not align with our results. In the present study, BDQ that was not pre-incubated at 37 °C displayed identical MIC values regardless of whether AA/P was added, indicating no detectable improvement in solubility or activity under our assay conditions.

It is important to emphasize that the behavior of BDQ in aqueous in vitro assays differs from its pharmaceutical stability profile. The marketed tablet formulation contains excipients [23,24,25,26,27] that maintain BDQ integrity and performance, whereas laboratory testing uses the pure active compound without these protective components. Our findings therefore apply exclusively to the microdilution assay environment and do not reflect or question the stability of the clinical product.

Taken together, the previous observations suggest that any interaction between AA/P and BDQ in our experimental setting does not reflect chemical stabilization, synergy or an improvement of solubility, but rather bacterial physiological changes.

Finally, while we acknowledge the limited number of isolates studied, the changes in the MICs were consistent across replicates. Future larger studies are necessary to define the variability and magnitude of BDQ instability under different assay conditions and to determine whether assay optimization, potentially including stabilizers, should be incorporated into routine laboratory practice.

## 4. Materials and Methods

### 4.1. Study Design

The MICs for 12 clinical isolates (eight MAC and four MTB) were determined. The antibiotics tested against MTB were BDQ, CFZ, MOX, PTM, LZD, and INH, while those tested against MAC included BDQ, CFZ, MOX, PTM, CLA, DLM, RIF and EMB. The study consisted of two phases: an initial screening to identify antibiotics with significant MIC changes after no incubation (0 days) and after pre-incubation for 7 days at 37 °C, followed by a stability assessment. In the stability phase, the antibiotics were also evaluated under two incubation conditions (0 days and 7 days at 37 °C), with and without stabilizers [11] (AA and P), using the microdilution method.

### 4.2. Isolate Selection

A total of 12 clinical isolates—eight NTM (four *M. avium* and four *M. intracellulare*) and four MTB—were collected from patients with chronic pulmonary obstructive disease, cystic fibrosis, and bronchiectasis and were analyzed in the Microbiology Department of the Hospital Clinic of Barcelona. All the isolates were maintained in *Mycobacterium* Growth Incubator Tube (Becton Dickinson, Sparks, MD, USA) media and Löwenstein-Jensen solid media (Becton Dickinson).

### 4.3. Antibiotic Selection

Newer anti-tuberculosis antibiotics (BDQ, PTM, DLM, and CFZ), as well as antibiotics used to treat MDR mycobacteriosis (LZD and MOX) and antibiotics routinely included in standard treatment regimens and combination studies (RIF, INH, EMB and CLA) were selected. For MTB isolates, the antibiotics tested were BDQ, CFZ, MOX, PTM, LZD and INH, while those tested for MAC isolates were BDQ, CFZ, MOX, PTM, CLA, DLM, RIF and EMB. AA and P, combined with each individual antibiotic, were chosen as potential antibiotic stabilizers [11].

### 4.4. Antibiotic and Stabilizer Preparation

The antibiotics BDQ, CFZ, PTM, DLM and LZD were purchased from Quimigen SL (Clinisciences Lab Solutions SL, Madrid, Spain), while MOX, INH, EMB, RIF, CLA, AA and P were purchased from Sigma Aldrich (Sigma-Aldrich, St. Louis, MO, USA). BDQ, CFZ, CLA, DLM, PTM, and RIF were diluted in dimethyl sulfoxide (DMSO) (Sigma Aldrich), whilst MOX, LZD, INH, and EMB were diluted in sterile distilled water. Both AA (Sigma Aldrich, St Louis, MO, USA) and P (Sigma Aldrich) stabilizers were dissolved in distilled water. Stock solutions of all antibiotics were prepared at a concentration of 10,000 μg/mL and diluted with sterile distilled water. The dilution for MOX and LZD was with DMSO and distilled water was used in the case of CFZ and PTM. Solutions of antibiotics in DMSO contained ≤2% of the solvent. Aliquots were stored at −20 °C until use, according to the guidelines for antimycobacterial drug susceptibility testing [16,17,18]. Each aliquot was thawed only once immediately prior to plate preparation and was not refrozen. No repeated freeze–thaw cycles were performed.

### 4.5. Pre-Incubation of the Antibiotics and Stabilizers

For the initial screening, all stock solutions of the antibiotics were prepared from the same lot to ensure consistency. Two aliquots of each antibiotic were prepared at working concentrations: one was immediately stored at −20 °C (0 days), and the other was pre-incubated at 37 °C for seven days (7 days) and thereafter at −20 °C. One week later, both aliquots were thawed and the MICs were determined using the microdilution method (Figure 1).

For antibiotics showing a variation in two or more dilutions in MIC values between the pre-incubated and non-pre-incubated conditions, a second lot was prepared for stability testing with and without stabilizers. In this case, the antibiotics were also prepared on the same day, using the same lot for all samples. For each antibiotic, four aliquots were prepared at working concentration: two containing the stabilizers, AA and P, and two without stabilizers (antibiotic with only its solvent). On the day of preparation, one aliquot with stabilizers and one without were immediately stored at −20 °C, while the remaining two aliquots (one with stabilizers and one without) were pre-incubated at 37 °C for seven days. After this incubation period, the pre-incubated aliquots were transferred to −20 °C, for storage. One week later, all four aliquots were thawed, and the MICs were determined using the microdilution method. Throughout this work, the antibiotics were designated according to their incubation conditions: 0 days of incubation (frozen immediately after preparation) and pre-incubation for 7 days at 37 °C, each tested with and without stabilizers (Figure 1).

### 4.6. Minimum Inhibitory Concentration Determination

The MICs were determined using the microdilution method. For each antibiotic incubated for 0 days and pre-incubated for 7 days as well as for AA and P alone and the combination of antibiotic with AA and P, the MIC was determined in 96-well plates (Smartech Biosciences, Barcelona, Spain) by adding 100 µL of Middlebrook 7H9 liquid medium (Becton Dickinson, Sparks, MD, USA) to each well. Then, 100 µL of antibiotic solution was added to the first well, and 2-fold serial dilutions ranging from 0.03 µg/mL to 8 µg/mL were performed. For solutions containing AA and P, the highest tested concentrations of these stabilizers were 3 mg/mL and 60 mg/mL, respectively. The stabilizer concentrations were defined according to the proportion of antibiotic dilution as described by Jikova et al. [11]. Finally, 100 µL of inoculum at a concentration of 1.5 × 10^5^ colony-forming units (CFU)/mL was added (1/1000 dilution of a 0.5 McFarland, using a nephelometer) (PhoenixSpec, Becton Dickinson, Sparks, MD, USA). For MTB isolates, the CFU clumps were disaggregated by repeated passage through an insulin syringe 15 to 20 times. The positive control wells contained 100 µL of Middlebrook 7H9 and 100 µL of inoculum. The negative control wells were also included by adding 200 µL of Middlebrook 7H9. All the microplates were incubated at 37 °C for 7 to 10 days until growth control was visible. After incubation, the plates were read using a Vizion System (Sensititre Vizion Digital MIC Viewing System, Thermo Fisher Scientific, Waltham, MA, USA). The MIC was interpreted as the lowest antibiotic concentration inhibiting mycobacterial growth. All BDQ stock solutions, pre-incubated aliquots, and MIC plates were handled and incubated under light-protected conditions [23] throughout the experiments. All the experiments were performed in triplicate.

Interpretation of Results: A significant reduction in MIC was defined as a decrease in two or more dilutions observed both between pre-incubated and non-pre-incubated antibiotics, and between antibiotics tested with stabilizers and the antibiotic alone.

### 4.7. Electrospray Ionization Mass Spectrometry (ESI-MS)

Samples for ESI-MS analysis were prepared to evaluate the stability of BDQ (*m*/*z* 555) under different incubation conditions. For each condition, three independent tubes were prepared and incubated at 37 °C for 0 or 7 days. The experimental groups included BDQ in powder form; BDQ solution; BDQ solution combined with AA and P; BDQ solution incubated with bacteria at a final concentration of 1.5 × 10^5^ CFU/mL; and BDQ solution combined with AA and P in the presence of bacteria. Samples containing bacteria were filtered through a 0.22 µm membrane filter prior to analysis.

ESI-MS analyses were performed at the Serveis Tècnics de Recerca of the Universitat de Girona with an Esquire 6000 mass spectrometer from Bruker Daltonics (Bremen, Germany) with a precision of ±0.2 *m*/*z*. The instrument was equipped with an electrospray ionization source, an ion trap analyzer, and an electron multiplier detector. It works in both positive and negative ionization mode, in a range of 50 to 3000 *m*/*z*. Samples were dissolved and they were introduced (5 μL) to the spectrometer through an Agilent Technologies 1200 Series HPLC automatic injector (Santa Clara, CA, USA) at a flow rate of 0.1 mL/min. Nitrogen was employed as drying and nebulizing gas. Results were analyzed with Bruker Compass DataAnalysis 4.0 software (Bruker Daltonics, Bremen, Germany). The experiments were performed in positive ionization mode.

## 5. Conclusions

The present study determined that BDQ undergoes in vitro degradation during extended incubation at 37 °C. This instability is effectively mitigated by the addition of AA and P, which preserve its antimicrobial activity in both MTB and MAC clinical isolates. In antibiotic susceptible tests that require further incubation, such as in time-kill assays for mycobacteria, or in a routine drug susceptibility test, we recommend adding AA and P to stabilize the activity of BDQ.

## Figures and Tables

**Table 1:** Minimum inhibitory concentrations (µg/mL) of clofazimine, linezolid, moxifloxacin, pretomanid and isoniazid, pre-incubated for 0 and 7 days, in four *M. tuberculosis* clinical isolates.

	ATB Pre-Incubation Days at 37 °C *	Total ATB Incubation Days at 37 °C **	MTB-ATCC	MTB-1	MTB-2	MTB-3
CFZ	0	7	0.125	0.25	0.25	0.25
	7	14	0.125	0.5	0.5	0.25
LZD	0	7	0.25	0.25	0.25	0.25
	7	14	0.25	0.25	0.5	0.25
MOX	0	7	0.25	0.25	0.25	0.25
	7	14	0.25	0.25	0.25	0.25
PTM	0	7	0.125	0.5	0.5	>2
	7	14	0.125	0.25	0.5	>2
INH	0	7	0.05	0.025	0.05	0.05
	7	14	0.05	0.025	0.05	0.05

PTM: pretomanid, CFZ: clofazimine, LZD: linezolid, MOX: moxifloxacin, INH: isoniazid, ATB: antibiotic, MTB-ATCC: *Mycobacterium tuberculosis* American Type Cell Culture, MTB: *Mycobacterium tuberculosis*. * Number of days the antibiotic was pre-incubated at 37 °C before MIC testing. ** Total number of days the antibiotic was exposed to 37 °C, including pre-incubation and MIC testing in the presence of the inoculum. The experiments were performed in triplicate.

**Table 2:** Minimum inhibitory concentrations (µg/mL) of clarithromycin, clofazimine, delamanid, ethambutol, moxifloxacin, pretomanid, and rifampicin pre-incubated for 0 and 7 days, in eight MAC clinical isolates.

	ATB Pre-Incubation Days at 37 °C *	Total ATB Incubation Days at 37 °C **	MAV-1	MAV-2	MAV-3	MAV-4	MIN-1	MIN-2	MIN-3	MIN-4
CLA	0	7	16	8	8	1	4	1	16	8
	7	14	16	8	8	2	8	2	16	8
CFZ	0	7	1	1	1	1	1	1	1	1
	7	14	1	1	1	1	1	1	1	1
DLM	0	7	32	64	32	32	32	8	32	32
	7	14	32	64	32	32	32	8	32	32
EMB	0	7	2	4	8	4	4	2	4	4
	7	14	2	4	8	4	4	2	4	4
MOX	0	7	2	4	0.5	<0.12	2	0.5	4	4
	7	14	2	4	0.5	<0.12	2	0.5	4	4
PTM	0	7	64	128	128	128	128	128	128	128
	7	14	64	128	128	128	128	128	128	128
RIF	0	7	0.25	0.125	0.125	0.125	<0.03	0.125	0.25	0.125
	7	14	0.25	0.125	0.125	0.125	<0.03	0.125	0.25	0.125

CLA: clarithromycin, CFZ: clofazimine, DLM: delamanid, EMB: ethambutol, MOX: moxifloxacin, PTM: pretomanid, RIF: rifampicin, ATB: antibiotic, MAV: *Mycobacterium avium*, MIN: *Mycobacterium intracellulare*. * Number of days the antibiotic was pre-incubated at 37 °C before MIC testing. ** Total number of days the antibiotic was exposed to 37 °C, including pre-incubation and MIC testing in the presence of the inoculum. The experiments were performed in triplicate.

**Table 3:** Minimum inhibitory concentrations (µg/mL) of bedaquiline, bedaquiline with stabilizers, and stabilizers alone, pre-incubated for 0 and 7 days, in four *M. tuberculosis*, four *M. avium* and four *M. intracellulare* clinical isolates.

	ATB Pre-Incubation Days *	Total ATB Incubation Days **	MTB-1	MTB-2	MTB-3	MTB-4	MAV-1	MAV-2	MAV-3	MAV-4	MIN-1	MIN-2	MIN-3	MIN-4
BDQ	0	7	0.01	0.03	0.06	0.03	0.06	0.06	0.03	0.125	0.03	0.03	0.03	0.03
	7	14	0.06	0.125	0.25	0.125	0.25	0.25	0.25	0.5	0.25	0.125	0.125	0.125
BDQ + AA + P	0	7	<0.01	0.01	0.03	0.01	0.06	0.06	0.03	0.125	0.03	0.03	0.03	0.03
	7	14	0.01	0.01	0.03	0.01	0.125	0.06	0.06	0.125	0.03	0.06	0.03	0.06
AA + P	0	7	>8	>8	>8	>8	>8	>8	>8	>8	>8	>8	>8	>8
	7	14	>8	>8	>8	>8	>8	>8	>8	>8	>8	>8	>8	>8

MTB: *Mycobacterium tuberculosis*, BDQ: bedaquiline, AA: ascorbic acid, P: pyruvate, ATB: antibiotic, MAV: *Mycobacterium avium*, MIN: *Mycobacterium intracellulare*. * Number of days the antibiotic was pre-incubated at 37 °C before MIC testing. ** Total number of days the antibiotic was exposed to 37 °C, including pre-incubation and MIC testing in the presence of the inoculum. The experiments were performed in triplicate.

## Data Availability

The original contributions presented in this study are included in the article. Further inquiries can be directed to the corresponding author(s).
